# Reproductive healthcare utilization for women in the sex trade: a qualitative study

**DOI:** 10.1186/s13584-024-00627-7

**Published:** 2024-09-02

**Authors:** Lior Birger, Yael Benyamini, Yael Goor, Zohar Sahar, Einat Peled

**Affiliations:** 1 Bob Shapell School of Social Work, Tel-Aviv University, Tel-Aviv, Israel; 2 The Ministry of Health, Jerusalem, Israel

**Keywords:** Reproductive health care, Maternity care, Childbirth, Pregnancy, Sex industry, Sex work, Sex trafficking

## Abstract

**Background:**

Women in the sex trade encounter significant challenges in obtaining reproductive healthcare. Reports of reproductive healthcare for women in the sex trade center on the prevention and termination of pregnancies, yet most women in the sex trade globally experience full term pregnancies and bear children. This study aimed to explore barriers and enabling factors to providing reproductive healthcare for women in the sex trade in Israel.

**Methods:**

We conducted a qualitative study utilizing a grounded theory method. Data were collected through semi-structured interviews, conducted between June 2021 and July 2022. Interviews were conducted with practitioners in healthcare settings (*n* = 20), practitioners in social services settings (*n* = 15), and women in the sex trade who received reproductive health care-related medical services (*n* = 13) in Israel. The interviews were audiotaped, transcribed, and thematically analyzed.

**Results:**

The findings indicated a multilayered structure of healthcare system-related factors and women-related factors. Stigma was noted as a multidimensional barrier, reflected in service providers’ attitude towards women in the sex trade, impairing the patient-provider relationship and impeding women’s help-seeking. However, the creation of a relationship of trust between the women and healthcare providers enabled better health outcomes.

**Conclusions:**

Based on the findings, we propose recommendations for designing and implementing reproductive healthcare services for women in the sex trade. The recommendations offer to (a) include women with lived experiences in planning and providing reproductive healthcare services, (b) adopt a trauma-informed approach, (c) emphasize nonjudgmental care, (d) train healthcare providers to reduce stigma and bias, and (e) enhance the affordability of health services for women experiencing marginalization.

## Background

Women in the sex trade face significant challenges in accessing reproductive health care (RHC) services. Their reproductive rights, including access to RHC services and information and the ability to make decisions concerning reproduction free of discrimination, are severely restricted. The term *women in the sex trade* [1] refers to women who exchange sex for money, housing or survival needs, and for whom different terminologies are commonly employed, such as prostitution, sex work and sex trafficking. RHC pertains to information, education, and services surrounding reproductive tract and sexually transmitted infections, abortion, family planning, prenatal care, safe births, and postnatal care [2]. Global data suggest that the incidence of pregnancy in general and unintended pregnancy in particular among women in the sex trade is high [3–5]. Paradoxically, while existing reports of RHC for women in the sex trade center on the prevention and termination of pregnancies, the majority of women in the sex trade globally are mothers [6]. Existing reports center on general healthcare, with little knowledge pertaining to RHC needs [7].

Prenatal care is essential to ensure maternal health of women in the sex trade and infant development, especially due to coinciding risk factors such as substance abuse, Sexually Transmitted Infections (STIs) and HIV, and to risks involved in working during pregnancy, including violence from men who pay for sex (MPS) [8]. The various risk factors may lead to poor pregnancy outcomes, such as higher rates of stillbirths and serious health problems [6]. Maternal prenatal stress may also lead to a range of short and long-term child physical and mental health problems [9]. The latter can impair attachment and parenting, and consequently also infant development [10].

However, access of women in the sex trade to health services is limited and thwarted by many barriers [11]. The bulk of available research in this domain, based on the perspectives of women regarding their utilization of general health services, is based on studies conducted mostly in lower-middle income countries [12]. Yet, many of the reported barriers were found across varied geographical locations, including high income countries [e.g., 13–15]. Of these barriers, stigma is the most prominent, as well as low education, ethnic/racial minority status, late discovery of the pregnancy [8], providers’ judgmental behavior [16], the setting of sex trade [15], lack of service information, social support, available and affordable services and healthcare policy, appointment scheduling problems, and quality of healthcare [16, 17]. Still, to date, no previous study explored how these experiences are shaped within the context of specific healthcare systems, such as the Israeli one.

The views of healthcare providers seem to play an important role in the access of women in of sex trade to health services and their utilization. Yet, there is a dearth of research on the experiences and perspectives of health professionals regarding helping women in the sex trade, with the exception of aid to victims of sex trafficking [18]. Moreover, supporting women in the sex trade involves unique considerations, yet little is offered in the way of evidence-based models of RHC to this population. Therefore, this qualitative Grounded Theory (GT) study aimed to explore barriers and enabling factors to the provision of RHC to women in the sex trade, in order to develop initial recommendations for best RHC practice, based on the perspectives and experiences of both women with lived experience and RHC professionals in Israel.

### The Israeli context

Israel has a robust, relatively efficient public healthcare system, yet disparities persist based on socioeconomic factors and a geographic maldistribution of resources [19]. Israeli women who trade sex are among the most marginalized populations in regard to healthcare in general and RHC in particular. In Israel, being paid for sex is not illegal as an adult, yet ‘prostitution’ is highly stigmatized [20]. In recent years, there is a noticeable shift in the Israeli public discourse toward viewing sex for pay as a form of violence against women [21], which resulted in a new law that criminalizes paying for sex — being enforced since 2020. Hence, being paid for sex is still not illegal, yet it is illegal for someone to pay for sex. It is estimated that about half of the women in the sex trade in Israel work at brothels and discrete apartments, whereas only 7% are involved in street prostitution [22]. The most updated survey, conducted in 2014, estimated the number of Israeli women in the sex trade at 10,000, of whom 70% are mothers to children, yet no data exist on their annual number of pregnancies and childbirths.

All Israeli citizens are entitled to universal public health services covered by the Israel National Health Insurance Law [19]. Thus, most Israeli women in the sex trade are entitled to free basic RHC services. Israeli national health insurance covers universal antenatal and postpartum health and mental health services. Terminations of pregnancy require approval from a pregnancy termination committee on specific grounds established by law. In practice, virtually all requests to terminate a pregnancy on these grounds are approved [23].

No data exist on the actual use of health services in general and RHC services in particular by women in the sex trade in Israel. While Israeli public RHC is generally of good coverage and quality, it is not available to non-citizens. Several specialized psychosocial support services for women in the sex trade in Israel have been established in recent years, mostly at the country’s geographic center, yet only two mobile clinics provide medical services to this population. According to a report of the Ministry of Welfare and Social Affairs [24], in 2022 an estimate of 2,533 women in the sex trade across Israel received specialized support services, 60% of them in the country’s geographical center. The most common support they received was related to food, clothing, hygiene and health needs. No data exists regarding the specific health-related needs that were addressed.

## Methods

### Sample and recruitment

This qualitative study utilized the GT method, aimed at developing data-embedded theoretical models, while using varied data sources to facilitate a multidimensional understanding of the studied phenomenon [25]. Data were collected through 48 semi-structured interviews with: (1) practitioners in social services directed at women in the sex trade (*n* = 15); (2) practitioners in healthcare settings (*n* = 20); and (3) women who trade sex in various settings, who received RHC-related medical services (*n* = 13).

As per GT *theoretical sampling* principles, the preliminary sampling plan was adjusted during the course of the study based on simultaneous data collection and analysis [25]. Convenience and snowball sampling of participants were based on the researchers’ personal and professional contacts and participants’ referrals [26]. We aimed at a sample that reflects heterogeneity in terms of geographical locations and demographic characteristics of the services and of women in the sex trade. A research assistant with lived experience assisted in developing the interview protocol and in the recruitment phase. Women in the sex trade were offered a gift card as an acknowledgment of their contribution.

### Data collection

All interviews were conducted between June 2021 and July 2022 by the first author and in Hebrew, except for one interview conducted by a research assistant in Arabic and translated to Hebrew. The interview protocol comprised questions regarding women’s experiences surrounding pregnancy and childbirth, covering RHC needs ranging from prevention to the postpartum phase. The questions focused on barriers and enabling factors to RHC utilization, and the relationship between service providers and women who trade sex. Twenty-five interviews were conducted via Zoom, 12 via phone, and 11 face-to-face, according to interviewees’ preferences. All interviews were recorded and transcribed verbatim.

### Participants

Women in the sex trade (*n* = 13). Women’s ages ranged from 21 to 40 (mean = 28). Ten were Jewish, and three were Arab women. All had young children who lived with them, except two who had partial custody over their child and were under child protection supervision. Two were pregnant at the time of the interview. Service providers (*n* = 35). All were women. Their ages ranged from 26 to 68 (mean = 44). Thirty-four were Jewish, one was Arab. Practitioners in social services directed at women in the sex trade included 11 social workers, two NGO directors, and two social advocates. Practitioners in healthcare settings included 12 social workers, two doulas, two obstetricians, a psychiatrist, an internal medicine doctor, an oncologist, and a nurse. Social services directed at women in the sex trade included state-funded and semi-funded organizations and NGOs, providing services for adults and young adults. Healthcare organizations included community HMOs and hospitals. The organizations offered services to a diverse population in terms of gender, sexual orientation, age, citizenship status, nationality, ethnicity, and setting of sex trade.

### Data analysis

Data analysis was conducted using MAXQDA software and followed the analytic steps of GT [25]. All data were coded using *open coding* to identify themes and concepts; *axial coding* included grouping and regrouping, resulting in *focused codes* that represented the most significant and frequent barriers and enabling factors. Finally, *theoretical coding* was used to organize the analysis into a coherent analytical framework. The entire research team discussed the analysis outcomes to deepen understanding and support credibility.

### Ethical considerations

The study was approved by [Tel Aviv University] University IRB. Extra care was taken to ensure the ethical tenet of “do no harm” in this sensitive study [27]. We made an effort to guarantee safe, private, and pleasant conditions for the interviews. The participants’ and organizations’ identifying information was omitted to maintain confidentiality.

## Results

The findings indicate a multi-layered structure of healthcare system-related and women-related barriers and enabling factors that are linked with the provision of RHC for women in the sex trade (see Table 1).

**Table 1:** Barriers and enabling factors to RHC utilization for women in the sex trade

	Healthcare system-related factors	Women-related factors
**Barriers**	Limited resources	Pressure from partner/MPS/pimp
	Limited accessibility	Trauma-related barriers
		Cultural barriers
	Lack of knowledge regarding women in the sex trade	Economic barriers
		Demographic barriers
	Burnout	Addictions
		Loneliness and isolation
	Bias and stigmatizing care	Lack of knowledge
		Fear of “systems”
	Providers’ identity	Perceptions regarding pregnancy
		Body perception
**Enabling factors**	Existing trauma-sensitive approach in health services	Accompanying person
	Inter-professional and inter-organizational cooperations	Women’s strengths
	A good patient-provider relationship	

### Barriers

#### Healthcare system-related barriers

***Limited resources***. Healthcare policy that resulted in limited staffing and long shifts negatively impacted the quality of care provided, especially for women with multiple needs (such as women with addictions). As indicated by a social worker in a hospital: *“In a [healthcare] system that is always on the brink of collapse*, *any event involving women with complex needs takes us out of balance.”*

***Limited accessibility***. The healthcare system was described as inaccessible by being ‘too bureaucratic’ and including long waiting times for appointments. *“It’s a 3-months wait for an appointment [with a gynecologist]. It doesn’t fit the needs of this population… they use medical services only when it’s already almost too late”* (Social workers, specialized service).

***Lack of knowledge regarding women in the sex trade***. Lack of acquaintance with health needs of women in the sex trade, lack of awareness of existing community-based services and lack of familiarity with women with lived experiences, all hindered adequate response of healthcare providers. An MD at a hospital said: “*Prostitution is really hard to comprehend*, *to imagine… doctors meet only few women who are rehabilitated*, *they don’t know what it looks like…*”.

***Burnout***. The combination of limited resources in the healthcare system, and the complex needs of (some) women in the sex trade, were described as contributing to personnel’s burnout, as manifested in practitioners’ stress and inability to provide good care. At times, these were accompanied by hostility that could be directed toward the women themselves. “*When treating a woman with complex needs*, *the staff gets burnt out*, *a shift becomes intolerable because the women that needs to go to the operation room*, *went out smoking… It creates hostility*” (Social worker, hospital).

***Bias and stigmatizing care***. Societal stigma towards women in the sex trade, often intersecting with other stigmas, such as addiction-related stigma and racism, was suggested to result in healthcare providers’ stigma expressed as feelings of rejection or aversion towards women, specifically towards those who are mothers. Such stigma – even if unintended – hinders providers’ ability to deliver good RHC to women in the sex trade. An activist in an organization for women in the sex trade said: “*There’s social stigma… It changes the way the staff looks at you*, *the tone of speech changes. As if it’s obvious that if you are in prostitution your judgment is questionable*”. Thus, some women encountered judgmental and condescending attitudes from providers, which were manifested verbally and physically. These included replacing a pregnant women’s doctor without notifying her, intervening in her decisions regarding childbirth, or speaking with the woman’s accompanying person “above her head”. At times, the fear of such stigmatizing care resulted in women’s attempts to conceal their being in the sex trade: “Approaching a professional [*in the healthcare system*] and saying that you are in prostitution and expecting a…normal, non-judging reaction? Wow, no way” (Woman with lived experience).

***Provider’s identity***. Providers’ ethnicity and gender impacted the nature of the patient-provider relationship and women’s service utilization. Receiving treatment from a male doctor was described as a primary barrier for women in the sex trade’s utilization of RHC. “*If a male gynecologist knows [that she is a prostitute]*, *some will take advantage of it and sexually harass her…thinking ‘oh*, *she is a prostitute*, *who would believe her?*‘” (Women with lived experience).

#### Women-related barriers

***Pressure from partner/MPS/pimp***. For some women in the sex trade who lived with a partner, physical or emotional violence prevented help-seeking, mainly around the decision to abort/continue the pregnancy. At times, women’s partners/MPS used physical power to try and abort the baby. Also, participants described the pressure from pimps as a barrier. For example, a social worker in a specialized service said: *“The pimp wants the woman to quickly abort so she can go back and continue to be her [the pimp’s] money maker.”*

***Trauma-related barriers***. Various traumatic experiences in women’s life – including sexual, emotional and physical violence in their childhood and while trading sex – were linked with women’s increased distrust in the healthcare system. “*The women’s ability to cooperate with the systems*, *the authorities… has been damaged throughout their lives - too many harms*, *too many breaches of confidence. So*, *they are already more suspicious*, *they disclose less to the staff… and then it creates a small circle of lies*, *which leads staff to be suspicious of them”* (Social worker, hospital). Also, complex PTSD (CPTSD) and its symptoms were seen as impeding some women’s ability to deal with bureaucracy. Trauma was linked with self-neglect, which caused some women to seek care only in emergency cases.

***Cultural barriers***. Socio-cultural taboos surrounding sex trading, pregnancy and childbirth served as a barrier for some women from ethno-national minority background. For example, due to the stigma surrounding sex trading in the Arab community, some Arab women had to conduct an abortion far from home. “*Arab women in the sex trade are afraid to go to their local HMO. This doctor treats her mother*, *grandmother*, *father – you can never know the level of confidentiality… they are afraid for their lives”* (Social worker, specialized service). Likewise, for some Eritrean asylum seekers, the taboo surrounding sex in general served as a barrier for acquiring knowledge on contraception.

***Economic barriers***. Poverty and the lack of access to bank accounts and credit cards due to existing debts and account confiscation, served as a prominent barrier. This manifested for example in the need to pay in cash for the pregnancy termination committee, or to pay the national health insurance via a standing order. This was exemplified in the account of a participant with lived experience: *“I didn’t go to all the pregnancy checkups*, *because they want money and I don’t have this money. So*, *I don’t go.”*

***Demographic barriers***. The intersecting identities of adolescent and young women in the sex trade were described as enhancing their difficulties in obtaining RHC, as well as living in the geographic periphery where good RHC was limited. *“Here it’s a periphery town*, *we have a very small pool of women gynecologists that we are willing to send our women to*, *knowing they are sensitive. Actually*, *there is just one such doctor.”* (Social worker, specialized service). Additionally, the lack of legal status for migrant and asylum-seeking women in the sex trade heavily limited RHC utilization. Unauthorized migrant women in the sex trade feared that approaching RHC services will cause their deportation.

***Addictions***. The findings revealed distinctive barriers for women with addictions. Being an active user was related to difficulties in planning ahead, delayed awareness of the pregnancy and irregular utilization of pregnancy services. *“[Women with addictions] don’t utilize health services so much. If it’s not an immediate need that interferes with survival – they postpone it.”* (MD, hospital). Similarly, a woman with lived experience noted regarding regular gynaecologist checkups: “Girls in that circle, they don’t go to checkups… they are only interested in what happens at the same moment, going to do that act, getting the money and buying their drugs”. This tendency had further negative consequences on the baby’s health, which required early detection and medical treatment. Women in the sex trade who had an addiction often left the hospital right after childbirth, thus depriving them and their babies from receiving adequate health care.

***Loneliness and isolation***. The lack of supportive relationships with family and friends often created loneliness and isolation. Practically, having no accompanying person prevented some women’s utilization of RHC services. This was described by a woman with lived experience: *“Late into the pregnancy*, *it becomes harder to go to checkups alone… I also have my concerns*, *I am anxious*, *I can’t handle stressful situations by myself”*. Moreover, loneliness was heightened due to women’s fear of disclosing their trading sex to family, friends, and service providers, and prevented postpartum help-seeking.

***Lack of knowledge***. Women who trade sex often lacked knowledge regarding sexual health, pregnancy and childbirth, as well as regarding RHC services. *“I was a bit traumatized by the first childbirth because I didn’t know what contractions are*, *and I didn’t know that I am in labor. Like*, *I thought it’s just normal pain”* (Woman with lived experience). This was linked by participants with late discovery of the pregnancy and with some women’s tendency to utilize Emergency Department services even when not necessary.

***Fear of “systems”***. Many women in the sex trade had previous negative experiences with the welfare system, including the placing of their and their relatives’ children out of home. Such negative experiences impacted their relationship with the healthcare system: women refrained from pregnancy checkups or did not report their emotional needs (e.g., postpartum depression) and health needs (e.g., drug use) for fear that their child be taken away. Non-disclosure might stand in the way of forming the trusted patient-provider relationship that enables the desirable natural development of childbirth. A doula participant explained: *The basic conditions for childbirth are that a woman will feel safe. And when there is a secret she is keeping*, *such as being in prostitution*, *and she is scared that it will be discovered*, *then the parasympathetic nervous system cannot take over… It can even lead to dissociation”*. This scenario was described as one that may lead to multiple medical interventions, which in turn increase the risk of postpartum depression.

***Perceptions regarding pregnancy***. Women’s perceptions of the pregnancy (as wanted, unwanted, or both) impacted RHC utilization. For example, women in the sex trade who view pregnancy as “a way out” of trading sex, may feel more anxious about their child being taken away, and therefore avoid RHC utilization. *“The women finally have something that is only theirs [the baby]*, *and they think this will help them exit this cycle… and the fear that they will take it from you*, *it’s very frightening”* (Women with lived experience). Alternatively, some women who continued trading sex while pregnant, linked their guilt about a potential harm to their infant caused by their sex trading while pregnant, with comprehensively utilizing RHC.

***Body perception***. Alienation and negative body perception – which some women in the sex trade linked with childhood abuse – along with fear of the pregnancy and the bodily changes it involves, hindered pregnancy discovery and checkups. “*We sometimes arrive at a “discrete” apartment and meet a pregnant woman*, *still working*, *in dissociation or denial of the pregnancy… and suddenly*, *7 or 8 months into the pregnancy she discovers she is pregnant*, *and its already a given fact*” (Social worker, specialized service).

### Enabling factors

#### Healthcare system-related factors

***Existing trauma-sensitive approach in healthcare services***. Some RHC services already implement a trauma-sensitive approach, which supports access and utilization of RHC by women in the sex trade. This was exemplified in the account of an MD working in a hospital: “*A woman’s request for a female gynecologist on the grounds of rape is already acceptable and would not look strange*”.

***Inter-professional and inter-organizational cooperation***. Existing cooperations were described as a prominent enabling factor.*“It really helps to have a contact person in the community… who makes sure the woman arrives [for checkups]*” (Social worker, hospital). Further, such cooperations were described as enhancing the continuity of care for women before and after childbirth.

***A good patient-provider relationship***. A relationship of trust and respect between women in the sex trade and healthcare providers enabled better health outcomes – for example when inserting an IUD, which could require women to attend more than one session. Such a close and trusted relationship was enhanced by providers’ reaching out to sex trading women, accessibility beyond fixed working hours, a non-judgmental attitude, and receiving care from a woman with lived experience. For example, a social worker in a specialized service described a positive experience with medical staff: “*The staff… stayed even after the closing hour… they didn’t judge her [the young women in the sex trade]*, *didn’t say ‘what’s with you? You’re delaying us*, *we need to go home’* “.

#### Women-related factors

***Accompanying person***. The presence of accompanying persons and good relations between them and healthcare providers, were described as enabling good RHC. Accompanying persons mentioned were family members or friends, but more often were members of specialized services for women in the sex trade. *“I had a doula*, *we met before the birth*, *she taught me a lot…and during childbirth I don’t know what I would have done without her…I was so scared and she gave me a lot of confidence”* (Women with lived experience).

***Women’s strengths***. Women’s own strengths and resilience supported their ability to seek and receive good RHC. A social worker in a specialized service noted this regarding a woman she assisted: *“She is a survivor*, *she can navigate her way*, *even in prostitution she didn’t get to the streets*, *she could handle herself”*. Such strengths manifested, for example, in women’s ability to mobilize others to assist them and women’s ability to learn about their rights and demand them.

## Discussion and recommendations

This qualitative study exposed multiple barriers and enabling factors to RHC utilization by women in the sex trade based on the experiences and perceptions of service providers and women who trade sex in Israel. These served as a base for developing evidence-based recommendations for best RHC practice with women in the sex trade, which could be implemented by professionals in health care settings in Israel, and is elaborated below.

The findings indicate that stigma is a central and multidimensional barrier to accessing good RHC for women in the sex trade: societal stigma impacts and is reflected in service providers’ attitude towards women in the sex trade, impairs providers’ quality of care and profoundly affects the nature of the patient-provider relationship. Furthermore, stigma may impede women’s decisions to seek help or prevent them from disclosing health needs related to trading sex. The results and their implications may be applicable to other contexts, as similar findings were reported in studies conducted in a wide range of low-, middle- and high-income countries, demonstrating the pervasive nature of stigma as encumbering the right of women in the sex trade to RHC [e.g., 28–30]. Even in New Zealand, where sex trading is decriminalized, research suggests that while most sex trading women have regular sexual health checkups, their checkups may be compromised since they hide their occupation from the GP [31].

The study provides several main contributions. First, it demonstrates the central role of a positive and supportive relationship between women in the sex trade and healthcare practitioners in facilitating good RHC surrounding pregnancy and childbirth. The ability to create such relations was enhanced when women had an accompanying person – typically a professional or a volunteer – especially when these were continuous relationships of trust. This points at the critical role of NGOs in enhancing sex trading women’s access to RHC services. Similar findings were reported in a UK-based study on general healthcare for street sex workers. In the study, sexual health services were rated by practitioners as the most accessible health services, due to their collaboration with charities who support women in the sex trade [32]. This calls for enhancing public RHC services’ cooperation with NGOs and other community services who may be better situated to create trusted relations with women in the sex trade. In contrast, as previously demonstrated in relation to other marginalized population in Israel, such as transgenders, discrimination and negative experiences in encountering healthcare providers may impair future healthcare utilization [33].

Second, the study reveals the unmet health, emotional and practical needs of women in the sex trade postpartum, which are rarely reported [12]. This gap is especially alarming given the increased risk of women in the sex trade for postpartum poor maternal mental health [34]. It should serve as a call for developing postpartum services that provide emotional and practical assistance. Such services should also be directed towards women who underwent abortions and stillbirths or gave birth but do not raise their children. Moreover, the study highlights the role of women’s perceptions of the welfare system in impairing their access to RHC, specifically, women’s fear of having their children taken to out-of-home placement. Such fear keeps some women in the sex trade from reporting their health needs, attending pregnancy checkups, or staying at the hospital after giving birth. Finally, it points to the salient role of the social determinants of health (SDH) in constructing unequal access to RHC by women in the sex trade [35]. SDH include the women’s childhood, geographical, economic, and cultural conditions shaped by unequal social policies and financial arrangements. Women in the sex trade may suffer from double or triple disadvantage, for example by belonging to an ethnic minority population or peripheral regions, which, as already demonstrated in the Israeli context, may by itself increase their health inequalities [36].

Based on the findings we offer recommendations for planning and implementing interventions surrounding RHC services for women in the sex trade in Israel. The recommendations address the healthcare system, offering general guidelines for policy makers and healthcare professionals in planning services and interventions, improving the accessibility of health services for women in the sex trade, and training personnel.

***General guidelines***. First, interventions should be based on woman-centered and trauma-informed approaches as well as principles of reproductive justice, which emphasize women’s preferences and life circumstances as a foundation for equitable RHC delivery [37]. Second, RHC utilization by women in the sex trade should be regarded as a “window of opportunity” to identify psychosocial needs surrounding violence, addictions, mental health, etc. The pregnancy termination committee and childbirth were highlighted as such opportunities, calling to include healthcare personnel working in those services in sensitization trainings (see below). When a woman in the sex trade is accompanied by another person, providers are advised to conduct a first intake with the women alone. Also, explanations regarding exploitation and available services should be posted in clinics, preferably in women’s public toilets, and in multiple languages. A third guideline calls to strengthen inter-organizational cooperation between healthcare providers and specialized services for women in the sex trade. Finally, we recommend including women with lived experiences in planning and providing RHC services for women in the sex trade.

***Enhancing health services’ accessibility***. Our research indicates two main complementary approaches that could direct policy makers within the Ministry of Health in improving RHC services’ accessibility. The first is improving accessibility to general health services. As many women in the sex trade do not disclose trading sex and thus avoid specialized services, improving general RHC services could indirectly benefit them. This can be done, for example, by enhancing community-based and free of charge childbirth preparation courses and doula services for marginalized women. A second, complementary approach includes developing, enhancing and widening the scope of existing specialized services for women in the sex trade, as many do utilize them. This may include expanding resources such as staff and medical check-ups within community-based services, offering as many treatments as possible in one place, free of charge, with walk-in services and flexible opening hours. A third recommendation – *“No woman gives birth alone”*, includes developing a structure for accompanying women in the sex trade during pregnancy, childbirth, and postpartum, and may entail expanding the availability of (free) doula services and offering physical and emotional postpartum support at women’s homes. Such structure could best be achieved through a collaboration between specialized services for women in the sex trade and the general healthcare system.

***Healthcare providers’ sensitization training***. Trainings should be directed at and offered to providers of reproductive health services across the country, including doctors, nurses, and social workers in hospitals (maternity, obstetrics and gynecology departments, women’s emergency department) as well as community health centers for women and state or municipal family health centers (“Tipat Halav”). It is advised that the National Social Work Services (NSWS), situated within the Ministry of Health (MoH), will be responsible for the development of the training module. This module could be integrated into or added to existing training on trauma-sensitive approaches and cultural and gender sensitive approaches to health care, which already exist within the MoH and were developed by the NSWS. The modules related to RHC of women in the sex trades could be offered by the MoH to community health centers for women and state or municipal family health centers and health maintenance organization.

Trainings should facilitate providers’ reflexivity on their perceptions regarding women in the sex trade, their pregnancy and childbirth, and provide knowledge regarding sex trade and existing services for women who trade sex. The latter includes information on the varied settings, intersecting identities and experiences of women in the sex trade; trauma; addictions and their implications for pregnancy and childbirth; identifying abuse; sexual health and health needs. Such manuals were already developed in other national contexts. For example, a comprehensive manual was developed by Brown et al. in South Africa [38]. It aims to support the sensitization of healthcare workers who provide services to marginalized groups, including sex workers. The manual is designated for individuals who already have a basic understanding and experience of health services provision. Evidence suggests that this sensitization training intervention with healthcare providers can increase providers’ empathy for sex workers and reduce stigmatizing and discriminatory judgements [39]. This manual, alongside the findings of the current study, could be adapted to the Israeli context and serve as a basis for training.

Indeed, training should aim to support providers’ adoption of a respectful and non-judgmental approach towards women in the sex trade, including sensitivity to the words used and the tone of speech. This also means recognizing women’s right for self-determination, their agency and choice: some women may not favor a trauma-informed approach, but rather prefer a focus on their medical needs in a non-judgmental manner.

A trauma-informed approach should serve as a leading guideline for training and include: supporting women’s utilization of existing trauma-informed birth centers in Israeli hospitals and preparation-for-birth tools; providing explanations before physical examinations; avoiding the presence of male personnel (if possible); guidelines on working with CPTSD symptoms such as dissociation; using mobile ultrasound devices instead of vaginal examinations in childbirth. Preferably, women with lived experience should co-lead or at least participate in such training.

The study has several limitations. First, although the literature suggests that the recommendations we developed could be implemented universally, the study was carried out in Israel and implementation of its recommendations to other settings should be done with attention to the local context. Regardless of the country in which these recommendations are adopted, it should be done with utmost sensitivity to the specific population at question. Second, the study focused on women in the sex trade with experience in utilizing RHC; the voices of sex trading women who refrain from utilizing RHC services are likely limited. Finally, the results focused on physical access to RHC and do not pertain to online utilization of RHC information and services.

## Conclusion

Our exploration of barriers and enabling factors to providing RHC to women in the sex trade revealed the salient role of stigma – compounded with other factors – as a barrier for women’s actualization of their reproductive rights. The practice implications contribute to existing recommendations for confidential, non-judgmental, trust building, adherence to principles of trauma-informed care, and cultural sensitivity [40–42], and for direct involvement of women in the sex trade in RHC interventions [41, 43]. This includes taking into account women’s diverse identities and self-determination. Aligning with previous studies, our findings call to emphasize the component of healthcare providers’ training. Training should include reflective aspects regarding providers’ perceptions of women in the sex trade, viewing these perceptions and biases as consciously and unconsciously impacting service delivery [44].

Finally, the study contributes to bridging RHC gaps for women in the sex trade, by enabling the inclusion of their perspectives in research about them and facilitating the adaptation of reproductive health services to their needs, with the hope of improving maternal health indicators of women in the sex trade and their children’s health trajectories.

## Data Availability

The datasets generated and/or analysed during the current study are not publicly available in order to maintain the anonymity of the participants.

## Abbreviations

RHC: Reproductive Health Care
GT: Grounded Theory
SDH: Social Determinants of Health
NSWS: National Social Work Services
MoH: Ministry of Health
STI: Sexually Transmitted Infection
MPS: Men who Pay for Sex
PTSD: Post Traumatic Stress Disorder
CPTSD: Complex Post Traumatic Stress Disorder
